# Preoperative Changes of Lung Nodule on Computed Tomography and Their Relationship With Pathological Outcomes

**DOI:** 10.3389/fsurg.2022.836924

**Published:** 2022-03-16

**Authors:** Shihong Zhou, Deng Cai, Chunji Chen, Jizhuang Luo, Rui Wang

**Affiliations:** Department of Thoracic Surgery, Shanghai Chest Hospital, Shanghai Jiao Tong University, Shanghai, China

**Keywords:** lung nodule, preoperative, follow-up, pathological outcomes, computed tomography

## Abstract

**Background:**

Whether changes of lung nodules on computed tomography could bring us helpful information related to their pathological outcomes remained unclear.

**Materials and Methods:**

This retrospective study was carried out among 1,185 cases of lung nodules in Shanghai Chest Hospital from January 2015 to April 2017, which did not shrink or disappear after preoperative follow-up over three months. Their imaging features, changes, and clinical characteristics were collected. A separate analysis was performed in nodules with or without growth in long-axis diameter after follow-up, searching significant changes related to nodule malignancy and the median interval of follow-up for reference. Further study was performed similarly in malignant nodules for discrimination of malignant grading.

**Results:**

Most nodules were stable (*n* = 885, 75%), whereas others grew (*n* = 300, 25%). For predicting nodule malignancy, increase in density (>10 Hounsfield units, median follow-up of 549 days) played an important role in growing group whereas it failed in stable group, and the increase in size was less significant in growing group. For discrimination of malignant grading, increase in density (>70 Hounsfield units, median follow-up of 366 days) showed its significance in stable group, and so did increase in size in growing group (maximum diameter growth >3.3 mm, median follow-up of 549 days, or average diameter growth >3.1 mm, median follow-up of 625 days).

**Conclusions:**

There were significant changes of lung nodules by follow-up on computed tomography, related to their pathological outcomes. The predictive power of increase in density or size varied in different situations, whereas all referred to a long-time preoperative follow-up.

## Introduction

Lung cancer is the leading cause of cancer-related death worldwide (1). Low-dose computed tomography (LDCT) lung screening trials were organized and performed in various countries, for the purpose of early diagnosis of lung cancer and therefore reducing mortality. The National Lung Screening Trial (NLST), the most famous one, showed a relative reduction of 20% in lung cancer mortality by LDCT (2). However, some lung nodules generated by benign diseases, such as infections and tuberculosis, have also been detected and then resected, which consequently leads to unnecessary medical costs and additional psychological anxiety (3). Nowadays, thin-section computed tomography (TSCT) has been widely used in clinical examination, which has made it possible to detect small nodules that may be the early stage of lung cancer, while at the same time stressing the concern of side effects of computed tomography (CT). Newly updated Fleischner Society Guidelines (4) recommended a follow-up interval of at least 3 months for nodules detected incidentally before resection. However, there was still some mist veiling the importance of preoperative follow-up on CT, which might be one possible reason for low guidelines adherence with pulmonologists (5).

In current practice, lung nodules identified by CT are managed according to the widely accepted guidelines that heavily rely on nodule size (4, 6–9). While recently researchers found that growth rate of nodules (10) and solid portions (11, 12) were also the risk factors of lung cancer, Lindell R. M. et al. clarified different growing patterns of lung nodules that were then diagnosed as lung cancer at last (13). These indicated that regarding to nodule malignancy, it was far from enough for judgment with only the consideration of nodule size. Changes of lung nodules through follow-up on CT, such as nodule growth and nodule density variation (which inferred solid portion variation), should also be considered before surgery. Moreover, it remains unclear whether the significance of these changes varied in different situations of discussing nodule malignancy and its grading. The purpose of our study was to carry out an analysis on predicting nodule malignancy and furthermore malignant grading, among lung nodules that grew and those without growth after follow-up individually, to find and compare the significance of nodule changes in different situations.

## Materials and Methods

### Patient Selection and Study Design

This retrospective study was approved by the institutional review board of Shanghai Chest Hospital, and written informed consent was waived. We reviewed the medical and radiological records of lung nodules detected on CT in our hospital from January 2015 to April 2017. Study subjects were enrolled only if (a) baseline and preoperative levels of CT results were available, and enrolled lung nodule was primary in every patient, which was smaller than 3 cm; (b) they had been followed up over 3 months before resection; (c) they were free of neoadjuvant therapy by any form; (d) they were resected after follow-up and their pathological outcomes were available; and (e) they did not shrink or disappear after follow-up. Clinical characteristics of patients were collected, including age, gender, smoking status, height, weight, and body mass index (BMI). Baseline level means the first time of nodule detection on CT, and preoperative level means the last time of CT scan just before nodule resection.

**Figure 1:**
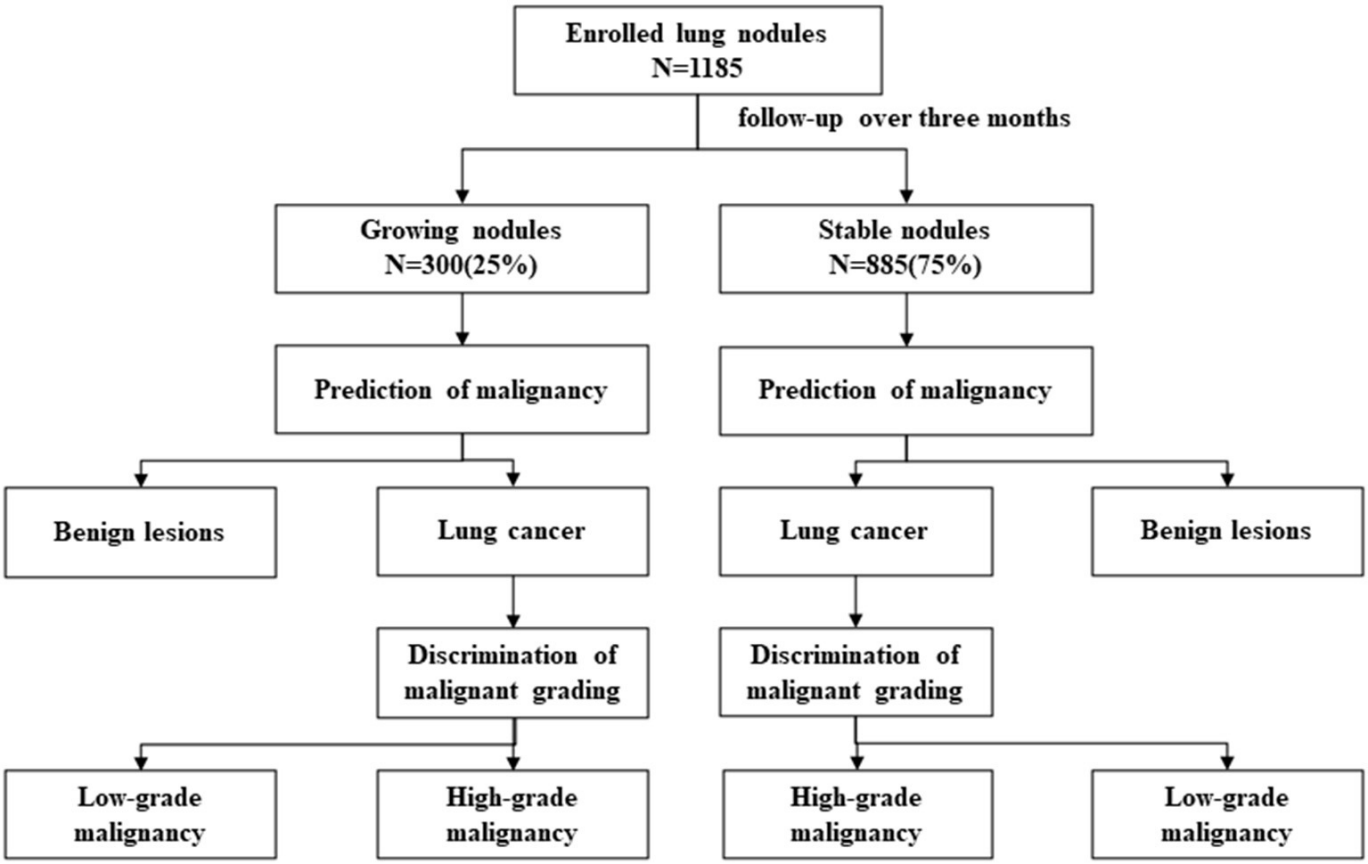
Flowchart of this study.

There were four major analyses in our study discussing about what actual role would changes of lung nodules play in the prediction of nodules' pathological outcomes: Analysis 1: prediction of nodule malignancy in growing nodules; Analysis 2: prediction of nodule malignancy in stable nodules; Analysis 3: discrimination of nodule malignant grading in growing malignant nodules; and Analysis 4: discrimination of nodule malignant grading in stable malignant nodules (Figure 1). Growing and stable nodules were divided according to whether they showed growth in long-axis diameter after follow-up over 3 months. Nodule malignancy and its grading were classified as “pathological classification” part said in the later section. We discussed the significance of changes of lung nodules in predicting nodule malignancy and furthermore malignant grading and compared them in different situations.

### CT and Nodule Measurements

Computed tomography scans were obtained with 128-detector row scanners (Brilliance, Philips, Cleveland, OH, USA) using the helical technique at the end of inspiration during one breath-hold. The scanning parameters were as follows: X-ray voltage, 120 kVp; tube current, 200 mA; pitch, 0.641; scan FOV, 400 mm.

All measurements were taken on the lung window setting (mean: −500 HU, width: 1,500 HU), on the CT section that displayed the largest nodule dimensions. Without the knowledge of pathological diagnosis, two radiologists having experience of at least 5 years independently evaluated the measurement of imaging parameters of 1,185 nodules, and any disagreement between their results was resolved by consensus. The long-axis diameter was measured as maximum diameter (MD), and then, the short-axis diameter was measured orthogonally to the long-axis as vertical maximum diameter (VMD). Nodule thickness (NT) was measured according to the CT section thickness and numbers of CT sections that nodule spanned. Average diameter (AD) was counted as the average of MD, VMD, and NT. We recorded nodules as “pure ground-glass nodule (PGGN)” if they contained no solid component but only ground-glass opacity (GGO), lesions of slightly increased CT attenuation through which the normal lung parenchyma structures, airways, and vessels are visually preserved (14), others as “mixed ground-glass nodule (MGGN)” if they were a combination of both GGO and solid portion, and “solid nodule (SN)” was composed entirely of the solid component. CT value was measured as the mean density of nodule in Hounsfield units. The consolidation-to-tumor ratio (CTR) was defined as the ratio of the long-axis diameter of consolidation and tumor. Both the ground-glass opacity and solid component were considered when the measurements were taken.

### Changes of Nodules on CT Scans

Growth was defined as an increase in long-axis diameter by at least 2 mm from baseline level (15) through follow-up, which was the divider of growing nodules and stable nodules. Changes of nodules on CT scans mainly included an increase in nodule size and an increase in nodule density in this study. On behalf of the increase in size, MD growth or AD growth was counted as the increase of MD or AD from baseline level to operative level, respectively. MD growth in ratio was the ratio of MD growth and MD, and AD growth in ratio was valued in the same way. Additionally, the volume doubling time (VDT) was also calculated based on the Schwartz formula: VDT = [t^*^log2]/[log Vt/V0], where “t” is the interval between follow-up on CT, “Vt” is the preoperative volume, and “V0” is the baseline volume (16). “V” is counted based on the formula: V = 4π(AD/2) ∧3/3. In line with the previous study (17), VDT was calculated only if the nodule volume changed by at least 25% from baseline level. On behalf of the increase in density, CT value increase was measured as the increase of nodule mean density in Hounsfield units from the baseline level.

### Pathological Classification

All enrolled lung nodules were resected after follow-up, and they were later diagnosed by a pathology specialist in our hospital after preparing formalin-fixed and paraffin-embedded (FFPE) specimens. Their pathological outcomes were recorded in our study, as shown in Table 1. The pathological classification of lung nodules was according to “*The 2015 World Health Organization (WHO) Classification of Tumors of the Lung, Pleura, Thymus, and Heart”* (18). Lung cancer was nodules clarified with malignancy. High-grade malignant nodules referred to nodules that were malignant and pathologically invasive, whereas low-grade malignant nodules were nodules that were malignant but free of pathological invasion.

**Table 1:** Pathological classification of lung nodules after follow-up.

**Pathological classification**	**Number**	**Malignant nodules**	**High-grade malignant nodules**
**Total**	1,185	1,059	391
**Adenocarcinoma**	1,034		
Invasive adenocarcinoma	356	**Yes**	**Yes**
*Lepidic adenocarcinoma*	*84*		
*Acinar adenocarcinoma*	*123*		
*Papillary adenocarcinoma*	*109*		
*Micropapillary adenocarcinoma*	*6*		
*Solid adenocarcinoma*	*16*		
*Other subtypes of invasive adenocarcinoma*	*18*		
Minimally invasive adenocarcinoma	347	**Yes**	No
Adenocarcinoma *in situ*	313	**Yes**	No
Atypical adenomatous hyperplasia	18	No	
**Squamous cell carcinoma**	16		
Keratinizing squamous cell carcinoma	9	**Yes**	**Yes**
Non-keratinizing squamous cell carcinoma	7	**Yes**	**Yes**
**Small cell carcinoma**	4	**Yes**	**Yes**
**Large cell neuroendocrine carcinoma**	6	**Yes**	**Yes**
**Carcinoid tumor(typical)**	3	**Yes**	No
**Large cell carcinoma**	3	**Yes**	**Yes**
**Adenosquamous carcinoma**	2	**Yes**	**Yes**
**Sarcomatoid carcinoma**	2	**Yes**	**Yes**
**Other and unclassified carcinoma**	2		
NUT carcinoma	1	**Yes**	**Yes**
Lymphoepithelioma-like carcinoma	1	**Yes**	**Yes**
**Adenoma**	3		
Sclerosing pneumocytoma	3	No	
**Mesenchymal tumor**	12		
Pulmonary hamartoma	11	No	
Epithelioid hemangioendothelioma	1	**Yes**	No
**Lymphohistiocytic tumors**	4		
MALT lymphoma	4	**Yes**	No
**Benign diseases**	94		
Infection	7	No	
Tuberculosis	14	No	
Interstitial fibrosis or hyperplasia	70	No	
Hemangioma	3	No	

*The number of diseases are in bold and the number of subtypes of these diseases are in italic*.

### Statistical Analysis

Continuous variables were examined by the Kolmogorov–Smirnov test, in which those according to normal distribution were analyzed with the independent sample *t*-test and described as “mean (standard deviation),” whereas others were analyzed with the Mann–Whitney *U* test and presented as “medians [IQRs].” Categorical variables were analyzed by the chi-squared test or Fisher's exact test, described as “frequency (proportion).” Receiver operating characteristic (ROC) curves were drawn to evaluate the predictive power of all factors, and the cutoff values were determined among significant factors where the Youden's indexes were at their maximums. Later, univariate logistic regression analyses in method of Enter were used to find the significant relative features. Additionally then, multivariate logistic regression analyses in method of Forward (LR) stepwise regression were used to find the independently significant covariates. Finally, these independently significant covariates were used to draw ROC curves together with their area under ROC curves (AUCs) and 95% confidence intervals (Cis) in four groups. All statistical tests were two-sided, with *p* < 0.05 considered statistically significant. Statistical analysis was done using IBM SPSS Statistics 26.0.

## Results

In total, 1,185 enrolled lung nodules included 885 (75%) stable nodules and 300 (25%) growing nodules (details of enrolled nodules are shown in Table 2). As for lung nodules diagnosed as lung cancer by pathological examination, there were 278 growing malignant nodules, whereas 781 malignant nodules were stable. More details of malignant nodules are listed in Table 2 and Supplementary Table 1.

**Table 2:** CT features, changes, and clinical characteristics of enrolled cases.

**Characteristics**	**Stable nodules**			**Growing nodules**		
	**Benign lesions**	**Malignant lesions**	**P**	**Benign lesions**	**Malignant lesions**	**P**
**Number**	104 (12%)	781 (88%)		22 (7%)	278 (93%)	
**Nodule type**			<0.001			0.008
PGGN	42 (40%)	639 (81%)		5 (23%)	137 (49%)	
MGGN	25 (24%)	114 (15%)		4 (18%)	65 (24%)	
SN	37 (36%)	28 (4%)		13 (59%)	76 (27%)	
**Nodule location**			0.002			0.637
Left upper lobe	16 (15%)	225 (29%)		6 (27%)	71 (26%)	
Left lower lobe	23 (22%)	93 (12%)		2 (9%)	38 (14%)	
Right upper lobe	34 (33%)	290 (37%)		10 (46%)	94 (34%)	
Right median lobe	10 (10%)	68 (9%)		0 (0%)	24 (9%)	
Right lower lobe	21 (20%)	105 (12%)		4 (18%)	51 (17%)	
**Baseline CT**						
Maximum diameter (mm)	7.9 [6.4, 10.0]	8.7 [6.9, 11.6]	0.003	12.2 [8.0, 16.4]	11.2 [7.8, 16.0]	0.945
Vertical maximum Diameter (mm)	6.0 [5.0, 7.6]	7.0 [5.5, 9.2]	0.001	9.4 [6.7, 13.2]	8.3 [6.0, 11.3]	0.337
Nodule thickness (mm)	7.0 [6.0, 10.0]	8.0 [6.0, 10.0]	0.002	10.0 [8.0, 19.0]	10.0 [7.7, 15.0]	0.715
Average diameter (mm)	6.9 [5.7, 9.1]	8.0 [6.4, 10.5]	0.001	10.8 [7.8, 15.3]	10.2 [7.3, 14.1]	0.663
CT value (HU)	−330 [−592, −32]	−576 [−656, −448]	<0.001	−59 [−341, 33]	−449 [−621, −159]	0.001
CTR	0.00 [0.00, 1.00]	0.00 [0.00, 0.00]	<0.001	1.00 [0.00, 1.00]	0.00 [0.00, 1.00]	0.003
**Preoperative CT**						
Maximum diameter (mm)	/	/	/	18.8 [11.5, 23.7]	16.0 [11.8, 22.9]	0.782
Vertical maximum diameter (mm)	/	/	/	12.3 [9.1, 16.4]	11.6 [8.2, 16.1]	0.405
Nodule thickness (mm)	/	/	/	15.0 [10.3, 23.5]	14.5 [10.0, 20.0]	0.565
Average diameter (mm)	/	/	/	16.1 [10.1, 21.7]	14.4 [10.5, 19.7]	0.596
CT value (HU)	−252 [−490, −2]	−552 [−634, −425]	<0.001	−37 [−403, 17]	−341 [−548, −30]	0.099
CTR	0.00 [0.00, 1.00]	0.00 [0.00, 0.00]	<0.001	1.00 [0.00, 1.00]	0.00 [0.00, 1.00]	0.004
**Change after follow–up**						
MD growth (mm)	/	/	/	4.5 [3.0, 8.0]	3.8 [2.7, 6.2]	0.152
MD growth in ratio (%)	/	/	/	50 [33, 70]	30 [22, 59]	0.132
AD growth (mm)	/	/	/	4.1 [2.4, 6.3]	3.0 [1.8, 5.3]	0.356
AD growth in ratio (%)	/	/	/	30 [23, 65]	30 [16, 54]	0.423
VDT (days)	/	/	/	223 [162, 482]	398 [201, 683]	0.118
CT value increase (HU)	13 [−13, 66]	17 [−18, 61]	0.945	−2 [−46, 40]	53 [3, 125]	0.003
**Patient characteristic**						
Age (years)	57 [47, 63]	58 [49, 64]	0.220	55 ± 10	63 ± 8	<0.001
Gender			0.032			0.123
Male	36 (35%)	192 (25%)		13 (59%)	116 (42%)	
Female	68 (65%)	589 (75%)		9 (41%)	162 (58%)	
Ever smoker			1.000			1.000
No	97 (93%)	729 (93%)		20 (91%)	242 (87%)	
Yes	7 (7%)	52 (7%)		2 (9%)	36 (13%)	
Height (cm)	162 [158, 168]	160 [158, 167]	0.255	169 [163, 175]	163 [158, 169]	0.003
Weight (kg)	62 [57, 70]	60 [54, 67]	0.009	67 [61, 71]	61 [55, 70]	0.046
BMI (kg/mm^2^)	23.78 (2.85)	23.11 (2.99)	0.032	23.40 [21.61, 24.63]	23.20 [21.30, 25.33]	0.757

*Data are n (%), median [IQR], or X (SD), and p-values are results of Mann–Whitney U test, chi-squared test, Fisher's exact test, or independent sample t-test*.

### Increase in Nodules' Density or Size After Follow-Up

Computed tomography value increase, another word as an increase in density, showed a significant discrepancy between growing nodules diagnosed as benign lesions and that diagnosed as lung cancer (Table 2), whereas it seemed to be nothing different between benign and malignant stable nodules (Table 2). However, in the case of malignant nodules only, CT value increase was quite different between stable nodules of low-grade malignancy and that of high-grade malignancy (Supplementary Table 1). CT value increase showed no significant difference between the two grades of malignancy among growing malignant nodules (Supplementary Table 1).

An increase in size was discussed among growing nodules, and no significant discrepancy was found between benign and malignant growing nodules (Table 2). Nevertheless, in the case of malignant nodules, MD growth, AD growth, AD growth in ratio, as well as VDT were clarified significantly different between growing nodules of low-grade malignancy and that of high-grade malignancy (Supplementary Table 1).

### Prediction of Nodule Malignancy in Growing Nodules

**Figure 2:**
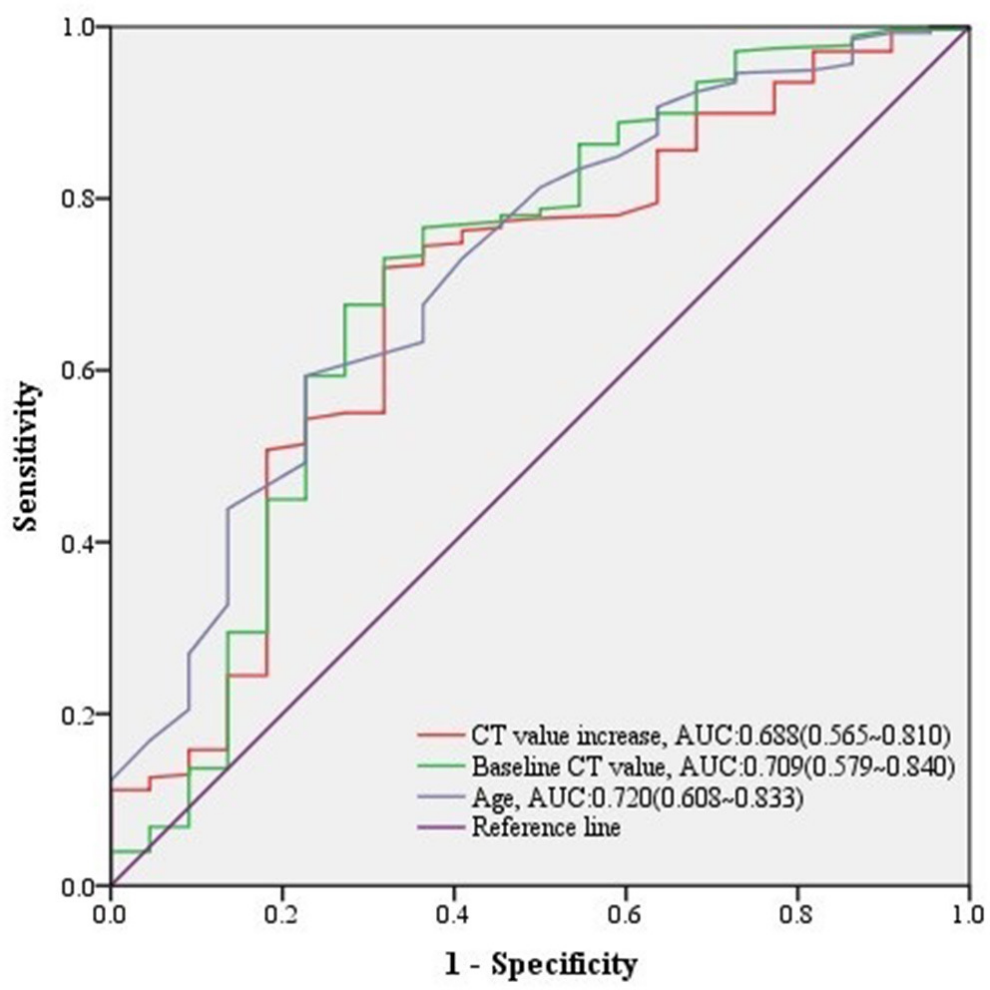
ROC curves of CT value increase and other predictors for malignant lesions in growing nodules. The predictive effect of significant covariates in growing nodules was presented in ROC curves, among which CT value increase showed a good performance predicting malignancy.

Among 300 growing nodules, baseline CT value, baseline CTR, preoperative CTR, and CT value increase, as well as age, height, and weight of patients were significant relative factors of nodule malignancy in univariate logistic regression analysis (Table 3). Baseline CT value, CT value increase, and age remained independent predictors in multivariate logistic regression analysis (Table 3). It revealed that CT value increase>10 HU after follow-up (*n* = 197, median = 549 days) (Table 4) was of significant value in predicting malignancy in growing nodules (AUC = 0.688, 95% CI: 0.565–0.810) (Figure 2).

**Table 3:** The univariate and multivariate analysis of predicting nodule malignancy in growing nodules.

**Characteristics**	**Identification of malignant lesions in growing nodules**					
	**Univariate logistic regression**			**Multivariate logistic regression**		
	**β**	**Odds ratio (95%CI)**	***P***	**β**	**Odds ratio (95%CI)**	***P***
**Baseline CT**						
CT value (>-191 HU)	−1.758	0.172 (0.068~0.439)	<0.001	−1.585	0.205 (0.077~0.546)	0.002
CTR (>0.80)	−1.345	0.260 (0.107~0.634)	0.003			
**Preoperative CT**						
CTR (>0.89)	−1.327	0.265 (0.109~0.646)	0.003			
**Change after Follow-up**						
CT value increase (>10 HU)	1.704	5.495 (2.158~13.989)	<0.001	1.393	4.029 (1.513~10.724)	0.005
**Patient Characteristic**						
Age (>62 years)	1.602	4.965 (1.780~13.843)	0.002	1.555	4.736 (1.625~13.808)	0.004
Height (>166 cm)	−1.292	0.275 (0.108~0.696)	0.006			
Weight (>63 kg)	−1.023	0.360 (0.142~0.910)	0.031			

*Only significant risk factors were listed*.

**Table 4:** The relationship between changes of lung nodules observed on CT and their pathological classification.

		**Stable nodules N, median (days)**				**Growing nodules N, median (days)**		
		**Benign lesions**	**Low-grade malignancy**	**High-grade malignancy**		**Benign lesions**	**Low-grade malignancy**	**High-grade malignancy**
CT value increase	≤ 0HU	42, 191	224, 181	60, 159	≤ 0 HU	12, 387	19, 301	46, 281
	>0HU, ≤ 70HU	36, 325	247, 250	80, 180	>0 HU, ≤ 10 HU	3, 160	8, 374	8, 227
	>70HU	26, 352	110, 308	***60, 366***	>10 HU	7, 499	***60, 582** ^***a***^*	***137, 532** ^***b***^*
MD growth					≤ 3.3 mm	8, 291	62, 368	57, 300
					>3.3 mm	14, 379	25, 903	***134, 549***
AD growth					≤ 3.1 mm	10, 317	72, 470	72, 242
					>3.1 mm	12, 387	15, 880	***119, 625***

^a, b^*Results of low-grade and high-grade growing malignant nodules; N, median (days) =197, 549*.

“*median (days)” means the interval of follow-up which was indicated with median days*.

*Lung nodules with significant changes were highlighted with bold and italic*.

### Prediction of Nodule Malignancy in Stable Nodules

As for 885 stable nodules, baseline and preoperative features were found significantly relative to malignant outcomes (Supplementary Table 2). However, CT value increase was found a lack of significance in prediction of nodule malignancy among stable nodules by either univariate or multivariate logistic regression analysis (Supplementary Table 2), inferring that it was quite the different case of predicting nodule malignancy in stable nodules or growing ones even if an increase in density was observed in both cases.

### Discrimination of Malignant Grading in Growing Malignant Nodules

**Figure 3:**
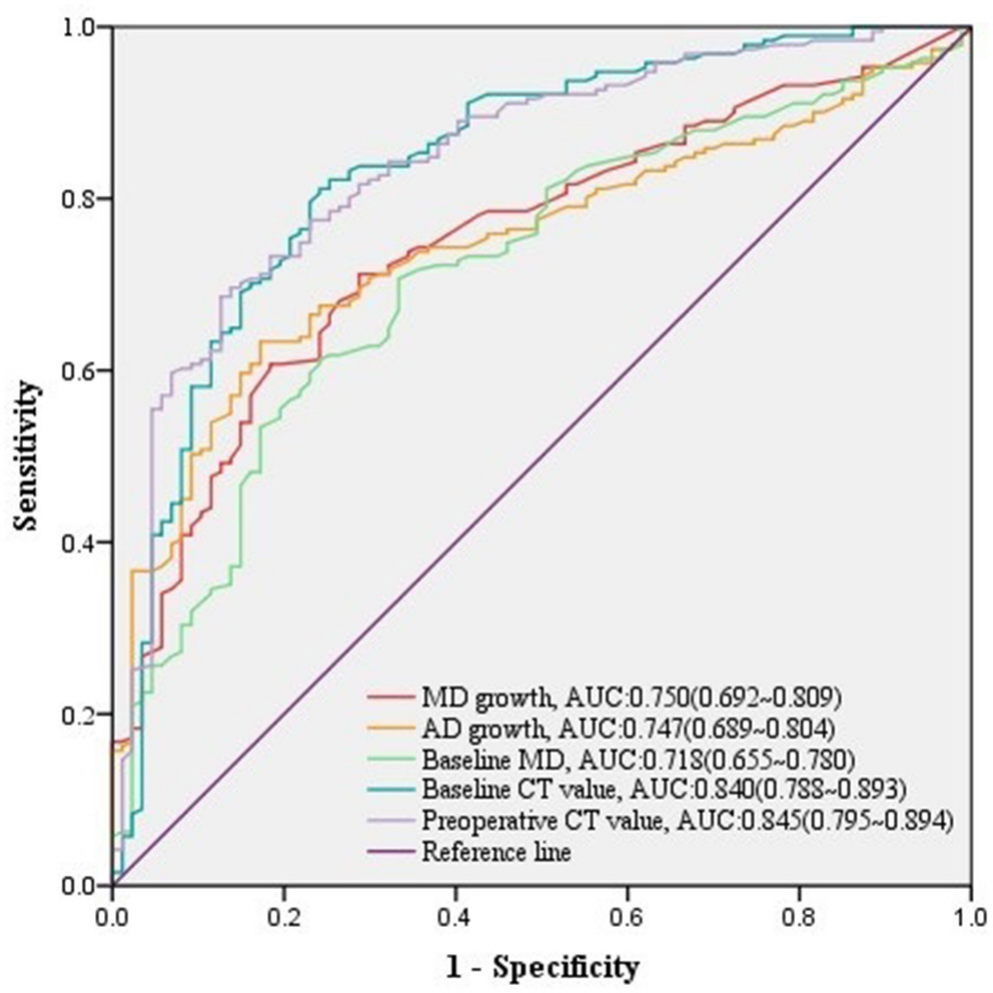
ROC curves of MD growth and AD growth and other predictors for high-grade malignancy in growing nodules. The predictive effect of significant covariates in growing malignant nodules was presented in ROC curves, among which either MD growth or AD growth showed a good performance in discriminating high-grade malignancy.

Among 278 growing malignant nodules, univariate logistic regression analysis revealed that nodule type, baseline MD, baseline VMD, baseline NT, baseline AD, baseline CT value, baseline CTR, preoperative MD, preoperative VMD, preoperative NT, preoperative AD, preoperative CT value, preoperative CTR, and MD growth, AD growth, AD growth in ratio, and VDT over follow-up, as well as gender and height of patients were significant relative factors of high-grade malignancy (Table 5). Whereas baseline MD, baseline CT value, preoperative CT value, MD growth, and AD growth remained independent predictors in multivariate logistic regression analysis, we could infer from it that changes of growing nodules, such as MD growth >3.3 mm (AUC = 0.750, 95% CI:0.692–0.809) after follow-up (*n* = 134, median = 549 days) (Table 4), as well as AD growth>3.1 mm (AUC = 0.747, 95% CI:0.689–0.804) after follow-up (*n* = 119, median = 625 days) (Table 4) were significant in predicting high-grade malignancy (Figure 3).

**Table 5:** The univariate and multivariate analyses of discriminating malignant grading in growing malignant nodules.

**Characteristics**	**Identification of high-grade malignancy in growing malignant nodules**					
	**Univariate logistic regression**			**Multivariate logistic regression**		
	**β**	**Odds ratio (95%CI)**	***P***	**β**	**Odds ratio (95%CI)**	***P***
**Nodule type**						
PGGN	0.000	1	<0.001			
MGGN	1.836	6.273 (2.955~13.317)	<0.001			
SN	3.022	20.531 (7.104~59.338)	<0.001			
**Baseline CT**						
Maximum diameter (>10.0 mm)	1.573	4.821 (2.799~8.305)	<0.001	1.992	7.328 (3.279~16.375)	<0.001
Vertical maximum diameter (>6.9 mm)	1.144	3.141 (1.852~5.328)	<0.001			
Nodule thickness (>10.5 mm)	1.893	6.642 (3.505~12.587)	<0.001			
Average diameter (>9.9 mm)	1.662	5.270 (3.004~9.246)	<0.001			
CT value (>-551 HU)	2.605	13.532 (7.349~24.915)	<0.001	2.032	7.631 (3.197~18.214)	<0.001
CTR (>0.53)	2.596	13.416 (4.721~38.128)	<0.001			
**Preoperative CT**						
Maximum diameter (>15.6 mm)	2.199	9.016 (4.847~16.770)	<0.001			
Vertical maximum diameter (>12.9 mm)	1.767	5.852 (3.090~11.081)	<0.001			
Nodule thickness (>11.7 mm)	2.589	13.323 (7.265~24.432)	<0.001			
Average diameter (>14.4 mm)	2.401	11.034 (5.697~21.372)	<0.001			
CT value (>-357 HU)	2.714	15.085 (7.474~30.445)	<0.001	1.709	5.521 (2.158~14.125)	<0.001
CTR (>0.47)	2.618	13.713 (4.826~38.965)	<0.001			
**Change after follow-up**						
MD growth (>3.3 mm)	1.814	6.132 (3.503~10.737)	<0.001	1.222	3.393 (1.322~8.709)	0.011
AD growth (>3.1 mm)	2.093	8.113 (4.325~15.216)	<0.001	1.203	3.329 (1.210~9.164)	0.020
AD growth in ratio (>50%)	1.340	3.820 (1.900~7.678)	<0.001			
VDT (>301 days)	−1.153	0.316 (0.173~0.576)	<0.001			
**Patient characteristic**						
Gender (Male)	0.971	2.641 (1.517~4.598)	0.001			
Height (>168 cm)	1.032	2.807 (1.516~5.194)	0.001			

*Only significant risk factors were listed*.

### Discrimination of Malignant Grading in Stable Malignant Nodules

**Figure 4:**
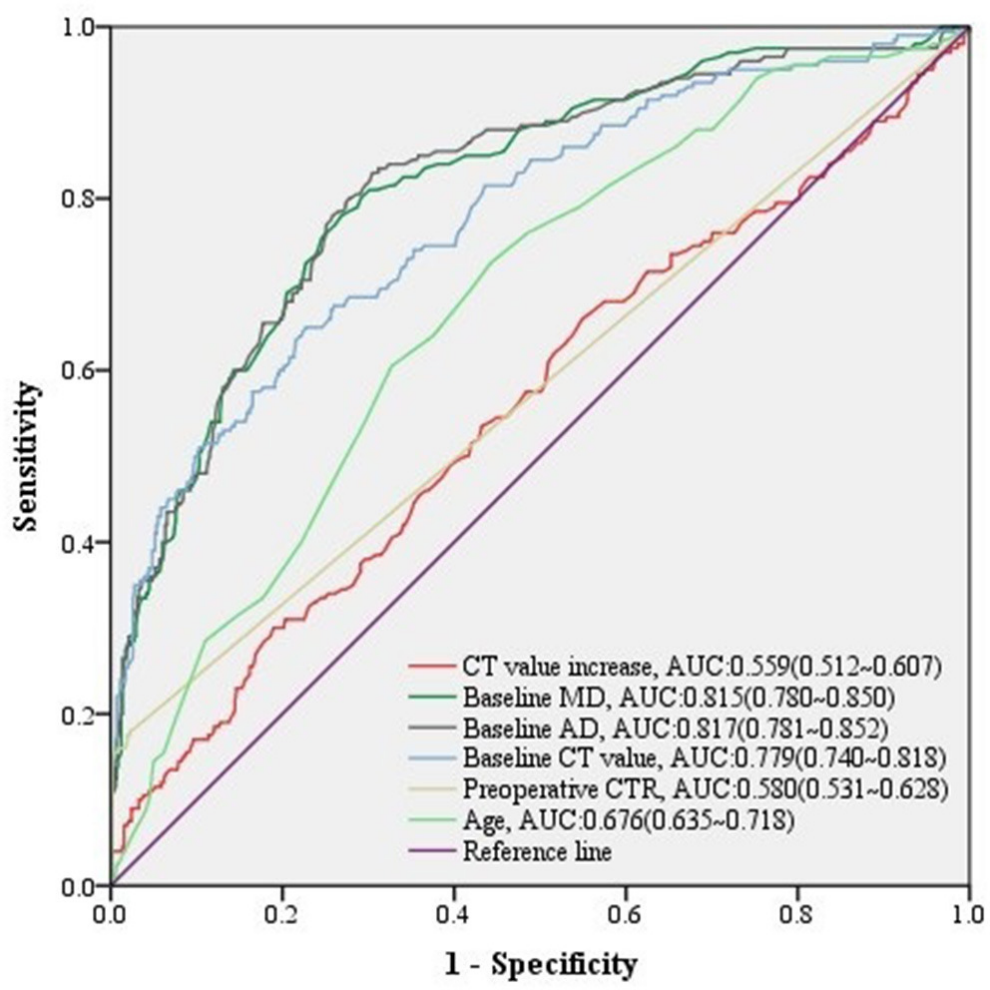
ROC curves of CT value increase and other predictors for high-grade malignancy in stable nodules. The predictive effect of significant covariates in stable malignant nodules was presented in ROC curves, among which CT value increase also showed a good performance discriminating high-grade malignancy.

Regarding to 781 stable malignant nodules, baseline MD, baseline VMD, baseline NT, baseline AD, baseline CT value, baseline CTR, preoperative CT value, preoperative CTR, and CT value increase from baseline level, as well as age, gender, height, and weight of patients were significant relative factors in predicting high-grade malignancy in univariate logistic analysis (Table 6). baseline MD, baseline AD, baseline CT value, preoperative CTR, and CT value increase, as well as age of patients remained independent predictors in multivariate logistic regression analysis (Table 6), we could infer from it that CT value increase>70 HU, was a significant change of stable nodules after follow-up (*n* = 60, median = 366 days) (Table 4) in predicting high-grade malignancy (AUC = 0.559, 95% CI:0.512–0.607) (Figure 4) among stable nodules.

**Table 6:** The univariate and multivariate analysis of discriminating malignant grading in stable malignant nodules.

**Characteristics**	**Identification of high-grade malignancy in stable malignant nodules**					
	**Univariate logistic regression**			**Multivariate logistic regression**		
	**β**	**Odds ratio (95%CI)**	**P**	**β**	**Odds ratio (95%CI)**	**P**
**Nodule Type**						
PGGN	0.000	1	<0.001			
MGGN	2.065	7.888 (5.130~12.129)	<0.001			
SN	4.934	138.894 (18.677~1,033.456)	<0.001			
**Nodule location**						
Left upper lobe	0.000	1	0.023			
Left lower lobe	0.114	1.121 (0.637~1.972)	0.692			
Right upper lobe	−0.014	0.986 (0.650~1.495)	0.946			
Right median lobe	0.490	1.632 (0.899~2.962)	0.108			
Right lower lobe	0.701	2.016 (1.218~3.338)	0.006			
**Baseline CT**						
Maximum diameter (>9.9 mm)	2.268	9.659 (6.595~14.147)	<0.001	1.108	3.029 (1.455~6.303)	0.003
Vertical maximum diameter (>8.0 mm)	1.906	6.723 (4.722~9.572)	<0.001			
Nodule thickness (>9.8 mm)	2.049	7.759 (5.390~11.169)	<0.001			
Average diameter (>8.5 mm)	2.411	11.144 (7.403~16.776)	<0.001	1.700	5.473 (2.546~11.764)	<0.001
CT value (>-505 HU)	1.843	6.317 (4.454~8.959)	<0.001	2.267	9.647 (6.022~15.456)	<0.001
CTR (>0.10)	2.185	8.890 (4.682~16.883)	<0.001			
**Preoperative CT**						
CT value (>-458 HU)	2.266	9.639 (6.694~13.878)	<0.001			
CTR (>0.27)	2.261	9.591 (4.969~18.511)	<0.001	1.390	4.015 (1.767~9.127)	0.001
**Change after follow-up**						
CT value increase (>70 HU)	0.607	1.835 (1.272~2.648)	0.001	1.297	3.657 (2.190~6.107)	<0.001
**Patient characteristic**						
Age (>58 years)	1.201	3.324 (2.340~4.721)	<0.001	0.608	1.837 (1.170~2.884)	0.008
Gender (Male)	0.803	2.232 (1.570~3.171)	<0.001			
Height (>167 cm)	0.604	1.829 (1.286~2.603)	0.001			
Weight (>73 kg)	0.764	2.146 (1.392~3.308)	0.001			

*Only significant risk factors were listed*.

## Discussion

In this study, we collected the details of follow-up CT data among lung nodules that did not shrink or disappear after follow-up over 3 months. We summarized significant changes of lung nodules by follow-up on CT which could predict their pathological outcomes and found that the predictive power of increase in density or size varied in different situations, whereas all referred to a long-time preoperative follow-up.

Nowadays, nodule size and growth rate remain the most widely used predictors to assess the probability of nodule malignancy, followed by nodule management according to the international guidelines (4, 7, 8, 19). Moreover, recent guidelines (4, 8) introduce nodule volume and VDT as the indicators of lung cancer. Several types of research have explored the predictive factors among CT quantitative parameters, radiomics features, and CT texture features to identify the invasiveness of lung nodules (11, 12, 20–23). However, it remained unclear what actual role would changes of lung nodules play in the prediction of nodules' pathological outcomes and whether they would always be significant in different situations, which was discussed in this article.

A plenty of previous researches (10, 13, 24, 25) have clarified that there are different growth patterns in lung cancer, among which some are stable whereas the others grow at fairly steady increments or demonstrated periods of accelerated growth. We presumed it a different case to talk about an increase in size and growth, and there might be other factors apart from growth that would show significant relevance with nodule malignancy and furthermore malignant grading. It needed further discussion with the ignorance of the confounding effect caused by nodule growth. Therefore, in our study, stepwise analyses were performed in different groups that grow nodules and stable ones, discussing prediction of nodule malignancy and furthermore malignant grading.

In this study, enrolled lung nodules were collected according to the inclusion and exclusion criteria, and we found that lung adenocarcinoma accounted for the majority of enrolled nodules compared with other lung diseases. Among them, invasive adenocarcinoma was sorted into high-grade malignant disease, whereas minimally invasive adenocarcinoma and adenocarcinoma *in situ* were classified as low-grade malignant diseases.

We noticed that CT value increase was a significant covariate associated with nodule malignancy in growing nodules, whereas we found that it failed to predict nodule malignancy in stable nodules. Nevertheless, it was still useful for predicting high-grade malignancy in stable malignant nodules. It was an interesting point of view that CT value increase, which might represent the increase of solid portion of nodules after follow-up, played different roles in the prediction of nodule pathological outcomes in different cases.

As for the increase in size, we found that MD growth and AD growth were of significant value in predicting high-grade malignancy after follow-up, whereas they seemed to fail in predicting nodule malignancy in growing nodules. Several studies had proved that lung nodules of high-grade malignancy, such as invasive adenocarcinoma, squamous cell carcinoma, and small cell carcinoma, tended to be an exponential increase in size (26, 27). These findings were in line with our study.

Regarding to lung nodules that were stable in size over 90 days in our study, there was no significant change of nodules on CT after follow-up predicting lung cancer, whereas we still found that baseline AD>7.2 mm, baseline CT value < -478 HU, and baseline CTR (consolidation-to-tumor ratio) <0.78, and preoperative CT value < -305 HU were significant predictors for lung cancer. Cruickshank et al. (28) concluded that the majority of solitary pulmonary nodules are benign with a small proportion representing early potentially curable lung cancer. Cohen et al. (29) indicated that part-solid nodules that persisted on a CT scan after 3 months had a high risk of malignancy compared to solid nodules. These might explain that nodules with lower CT value or lower CTR are more possible to be lung cancer. Moreover, larger AD was strongly associated with lung cancer, which was in line with the international management (4, 7, 8, 19) and the statement of Wang et al. (30). Moreover, discrepancy found in predicting nodule malignancy between stable and growing nodules inferred that it was a different situation for discussing the meaningful changes of stable nodules and growing nodules.

Nodules that shrunk after follow-up were excluded from discussion in our study, because they tended to be infections or other benign lesions. There was a large part of stable nodules in our study, which were mainly the early stage of lung adenocarcinoma. Therefore, their slow change was expected. Stable nodules might be resected in our hospital because of an increase in density, bad outcomes of other preoperative examination (such as serum tumor markers testing, PET/CT screening), or patients' will. In our study, we found that it was not a good idea to resect stable nodules decisively by the proof of increase in density, which was lack of significance in such prediction.

Nevertheless, our study possessed some limitations. First, our research was a retrospective study, as such, with a possibility of selection bias. Second, the distribution of cases in our study was not balanced, and there were relatively less cases in benign lesions. Third, our study did not investigate the prognosis of enrolled cases; thus, further study is needed. In this study, we aimed to discuss the relationship between changes of lung nodules that happened on follow-up CT scans and their pathological outcomes. Enrolled nodules were all scanned on CT at least two times and were finally resected and examined for pathological diagnosis. Lung nodules scanned on CT only once or those which were not resected after follow-up were excluded due to their insufficient data for discussion. Therefore, relatively less cases diagnosed as benign lesions were possible.

In conclusion, significant changes of lung nodules by follow-up on CT, related to their pathological outcomes, were found in our study. An increase in density and increase in size were proved to be significant covariates associated with nodule pathological outcomes. The practical value of increase in density or size varied in different situations, whereas all referred to a long period of preoperative follow-up.

## Data Availability Statement

The raw data supporting the conclusions of this article will be made available by the authors, without undue reservation.

## Author Contributions

SZ: conception and design. RW: administrative support, responsible for the conception and design of the study, and provided material support and administrative support. DC: provision of study materials or patients. CC: collection and assembly of data. JL: data analysis and interpretation. All authors: manuscript writing and final approval of manuscript.

## Funding

This work was supported by the National Natural Science Foundation of China (Grant Number 81773007).

## Conflict of Interest

The authors declare that the research was conducted in the absence of any commercial or financial relationships that could be construed as a potential conflict of interest.

## Publisher's Note

All claims expressed in this article are solely those of the authors and do not necessarily represent those of their affiliated organizations, or those of the publisher, the editors and the reviewers. Any product that may be evaluated in this article, or claim that may be made by its manufacturer, is not guaranteed or endorsed by the publisher.

## Abbreviations

CT: computerized tomography
ROC: receiver operating characteristic
AUC: area under ROC curve
CI: confidence interval
LDCT: low-dose computed tomography
NLST: National Lung Screening Trial
TSCT: thin-section CT
BMI: body mass index
MD: maximum diameter
VMD: vertical maximum diameter
NT: nodule thickness
AD: average diameter
GGO: ground-glass opacity
PGGN: pure ground-glass nodule
MGGN: mixed ground-glass nodule
SN: solid nodule
CRT, C/T ratio: consolidation-to-tumor ratio
VDT: volume doubling time
IQR: interquartile range.

## References

[1] National Lung Screening Trial Research T. Lung cancer incidence and mortality with extended follow-up in the national lung screening trial. J Thorac Oncol. (2019) 14:1732–42. 10.1016/j.jtho.2019.05.044 31260833 PMC6764895

[2] National Lung Screening Trial Research T Aberle DR Adams AM Berg CD Black WC Clapp JD. Reduced lung-cancer mortality with low-dose computed tomographic screening. N Engl J Med. (2011) 365:395–409. 10.1056/NEJMoa1102873 21714641 PMC4356534

[3] Anderson IJ Davis AM. Incidental pulmonary nodules detected on CT images. JAMA. (2018) 320:2260–1. 10.1001/jama.2018.16336 30419095

[4] MacMahon H Naidich DP Goo JM Lee KS Leung ANC Mayo JR. Guidelines for management of incidental pulmonary nodules detected on CT images: from the fleischner society 2017. Radiology. (2017) 284:228–43. 10.1148/radiol.2017161659 28240562

[5] Bueno J Landeras L Chung JH. Updated fleischner society guidelines for managing incidental pulmonary nodules: common questions and challenging scenarios. Radiographics. (2018) 38:1337–50. 10.1148/rg.2018180017 30207935

[6] MacMahon H Austin JH Gamsu G Herold CJ Jett JR Naidich DP. Guidelines for management of small pulmonary nodules detected on CT scans: a statement from the Fleischner Society. Radiology. (2005) 237:395–400. 10.1148/radiol.2372041887 16244247

[7] Naidich DP Bankier AA MacMahon H Schaefer-Prokop CM Pistolesi M Goo JM. Recommendations for the management of subsolid pulmonary nodules detected at CT: a statement from the Fleischner Society. Radiology. (2013) 266:304–17. 10.1148/radiol.12120628 23070270

[8] Callister ME Baldwin DR Akram AR Barnard S Cane P Draffan J. British Thoracic Society guidelines for the investigation and management of pulmonary nodules. Thorax. (2015) 70:ii1–ii54. 10.1136/thoraxjnl-2015-207168 26082159

[9] Wood DE. National Comprehensive Cancer Network (NCCN) clinical practice guidelines for lung cancer screening. Thorac Surg Clin. (2015) 25:185–97. 10.1016/j.thorsurg.2014.12.003 25901562

[10] Zhang R Tian P Qiu Z Liang Y Li W. The growth feature and its diagnostic value for benign and malignant pulmonary nodules met in routine clinical practice. J Thorac Dis. (2020) 12:2019–30. 10.21037/jtd-19-3591 32642104 PMC7330364

[11] Yanagawa M Kusumoto M Johkoh T Noguchi M Minami Y Sakai F. Radiologic-pathologic correlation of solid portions on thin-section CT images in lung adenocarcinoma: a multicenter study. Clin Lung Cancer. (2018) 19:e303–e12. 10.1016/j.cllc.2017.12.005 29307591

[12] Yamada N Kusumoto M Maeshima A Suzuki K Matsuno Y. Correlation of the solid part on high-resolution computed tomography with pathological scar in small lung adenocarcinomas. Jpn J Clin Oncol. (2007) 37:913–7. 10.1093/jjco/hym133 18211981

[13] Lindell RM Hartman TE Swensen SJ Jett JR Midthun DE Mandrekar JN. 5-year lung cancer screening experience: growth curves of 18 lung cancers compared to histologic type, CT attenuation, stage, survival, and size. Chest. (2009) 136:1586–95. 10.1378/chest.09-0915 19581354 PMC2789925

[14] Pedersen JH Saghir Z Wille MM Thomsen LH Skov BG Ashraf H. Ground-glass opacity lung nodules in the era of lung cancer CT screening: radiology, pathology, and clinical management. Oncology. (2016) 30:266–74. 26984222

[15] Hiramatsu M Inagaki T Inagaki T Matsui Y Satoh Y Okumura S. Pulmonary ground-glass opacity (GGO) lesions-large size and a history of lung cancer are risk factors for growth. J Thorac Oncol. (2008) 3:1245–50. 10.1097/JTO.0b013e318189f526 18978558

[16] Schwartz M A. biomathematical approach to clinical tumor growth. Cancer. (1961) 14:1272–94. 10.1002/1097-0142(196111/12)14:6&lt;1272::AID-CNCR2820140618&gt;3.0.CO;2-H 13909709

[17] van Klaveren RJ Oudkerk M Prokop M Scholten ET Nackaerts K Vernhout R. Management of lung nodules detected by volume CT scanning. N Engl J Med. (2009) 361:2221–9. 10.1056/NEJMoa0906085 19955524

[18] Travis WD Brambilla E Nicholson AG Yatabe Y Austin JHM Beasley MB. The 2015 world health organization classification of lung tumors: impact of genetic, clinical and radiologic advances since the 2004 classification. J Thorac Oncol. (2015) 10:1243–60. 10.1097/JTO.000000000000063 26291008

[19] Gould MK Donington J Lynch WR Mazzone PJ Midthun DE Naidich DP. Evaluation of individuals with pulmonary nodules: when is it lung cancer? Diagnosis and management of lung cancer, 3rd ed: American College of Chest Physicians evidence-based clinical practice guidelines. Chest. (2013) 143:e93S–e120S. 10.1378/chest.12-2351 23649456 PMC3749714

[20] Shikuma K Menju T Chen F Kubo T Muro S Sumiyoshi S. Is volumetric 3-dimensional computed tomography useful to predict histological tumour invasiveness? Analysis of 211 lesions of cT1N0M0 lung adenocarcinoma. Interact Cardiovasc Thorac Surg. (2016) 22:831–8. 10.1093/icvts/ivw037 26920725 PMC4986781

[21] Wang B Tang Y Chen Y Hamal P Zhu Y Wang T. Joint use of the radiomics method and frozen sections should be considered in the prediction of the final classification of peripheral lung adenocarcinoma manifesting as ground-glass nodules. Lung Cancer. (2020) 139:103–10. 10.1016/j.lungcan.2019.10.031 31760351

[22] Li X Zhang W Yu Y Zhang G Zhou L Wu Z. CT features and quantitative analysis of subsolid nodule lung adenocarcinoma for pathological classification prediction. BMC Cancer. (2020) 20:60. 10.1186/s12885-020-6556-6 31992239 PMC6986053

[23] Chae HD Park CM Park SJ Lee SM Kim KG Goo JM. Computerized texture analysis of persistent part-solid ground-glass nodules: differentiation of preinvasive lesions from invasive pulmonary adenocarcinomas. Radiology. (2014) 273:285–93. 10.1148/radiol.14132187 25102296

[24] Heuvelmans MA Vliegenthart R de Koning HJ Groen HJM van Putten M Yousaf-Khan U. Quantification of growth patterns of screen-detected lung cancers: the NELSON study. Lung Cancer. (2017) 108:48–54. 10.1016/j.lungcan.2017.02.021 28625647

[25] Lee HJ Goo JM Lee CH Yoo CG Kim YT Im JG. Nodular ground-glass opacities on thin-section CT: size change during follow-up and pathological results. Korean J Radiol. (2007) 8:22–31. 10.3348/kjr.2007.8.1.22 17277560 PMC2626694

[26] Henschke CI Yankelevitz DF Yip R Reeves AP Farooqi A Xu D. Lung cancers diagnosed at annual CT screening: volume doubling times. Radiology. (2012) 263:578–83. 10.1148/radiol.12102489 22454506 PMC3329268

[27] Oda S Awai K Murao K Ozawa A Utsunomiya D Yanaga Y. Volume-doubling time of pulmonary nodules with ground glass opacity at multidetector CT: assessment with computer-aided three-dimensional volumetry. Acad Radiol. (2011) 18:63–9. 10.1016/j.acra.2010.08.022 21145028

[28] Cruickshank A Stieler G Ameer F. Evaluation of the solitary pulmonary nodule. Intern Med J. (2019) 49:306–15. 10.1111/imj.14219 30897667

[29] Cohen JG Reymond E Lederlin M Medici M Lantuejoul S Laurent F. Differentiating pre- and minimally invasive from invasive adenocarcinoma using CT-features in persistent pulmonary part-solid nodules in Caucasian patients. Eur J Radiol. (2015) 84:738–44. 10.1016/j.ejrad.2014.12.031 25623825

[30] Wang H Weng Q Hui J Fang S Wu X Mao W. Value of TSCT features for differentiating preinvasive and minimally invasive adenocarcinoma from invasive adenocarcinoma presenting as subsolid nodules smaller than 3 cm. Acad Radiol. (2020) 27:395–403. 10.1016/j.acra.2019.05.005 31201034

