# Supplementary material for: Preoperative Changes of Lung Nodule on Computed Tomography and Their Relationship With Pathological Outcomes
Source: Front Surg. 2022 Mar 16;9:836924. doi: 10.3389/fsurg.2022.836924 (PMC8965753; doi:10.3389/fsurg.2022.836924)
Supplement: Supplementary file 1 [file Data_Sheet_1.docx]

**E-Tables**

e-Table 1: CT features, changes and clinical characteristics of malignant nodules.

| ***Characteristics*** | ***Stable Malignant Nodules*** | | ***P*** | ***Growing Malignant Nodules*** | | ***P*** |
| --- | --- | --- | --- | --- | --- | --- |
|  | ***Low-grade Malignancy*** | ***High-grade Malignancy*** |  | ***Low-grade Malignancy*** | ***High-grade Malignancy*** |  |
| **Number** | 581(74%) | 200(26%) |  | 87(31%) | 191(69%) |  |
| **Nodule Type** |  |  | <0.001 |  |  | <0.001 |
| PGGN | 535(92%) | 104(52%) |  | 73(84%) | 64(34%) |  |
| MGGN | 45(8%) | 69(34%) |  | 10(11%) | 55(29%) |  |
| SN | 1(0%) | 27(14%) |  | 4(5%) | 72(37%) |  |
| **Nodule Location** |  |  | 0.036 |  |  | 0.056 |
| Left upper lobe | 174(30%) | 51(25%) |  | 21(24%) | 50(26%) |  |
| Left lower lobe | 70(12%) | 23(12%) |  | 6(7%) | 32(17%) |  |
| Right upper lobe | 225(39%) | 65(32%) |  | 37(43%) | 57(30%) |  |
| Right median lobe | 46(8%) | 22(11%) |  | 10(11%) | 14(7%) |  |
| Right lower lobe | 66(11%) | 39(20%) |  | 13(15%) | 38(20%) |  |
| **Baseline CT** |  |  |  |  |  |  |
| Maximum Diameter (mm) | 8.0[6.5, 10.0] | 12.5[10.1, 17.7] | <0.001 | 8.7[6.7, 11.2] | 13.1[9.1, 18.6] | <0.001 |
| Vertical Maximum Diameter (mm) | 6.4[5.2, 7.9] | 9.6[7.4, 13.1] | <0.001 | 6.5[5.6, 9.4] | 9.0[6.6, 12.4] | <0.001 |
| Nodule Thickness (mm) | 8.0[6.0, 10.0] | 11.0[9.0, 15.0] | <0.001 | 8.0[6.0, 10.0] | 12.0[10.0, 16.0] | <0.001 |
| Average Diameter (mm) | 7.3[6.0, 9.0] | 11.1[9.1, 15.4] | <0.001 | 7.9[6.2, 10.2] | 11.5[8.4, 16.1] | <0.001 |
| CT value (HU) | -607[-678, -513] | -397[-573, -203] | <0.001 | -637[-700, -557] | -316[-513, -34] | <0.001 |
| CTR | 0.00[0.00, 0.00] | 0.00[0.00, 0.00] | <0.001 | 0.00[0.00, 0.00] | 0.00[0.00, 1.00] | <0.001 |
| **Preoperative CT** |  |  |  |  |  |  |
| Maximum Diameter (mm) | / | / | / | 11.8[9.3, 15.0] | 18.9[13.8, 26.0] | <0.001 |
| Vertical Maximum Diameter (mm) | / | / | / | 9.0[7.1, 11.9] | 13.4[9.7, 18.0] | <0.001 |
| Nodule Thickness (mm) | / | / | / | 10.0[8.0, 12.0] | 17.0[12.5, 22.0] | <0.001 |
| Average Diameter (mm) | / | / | / | 10.5[8.3, 12.6] | 17.0[12.2, 21.4] | <0.001 |
| CT value (HU) | -582[-652, -488] | -340[-523, -121] | <0.001 | -576[-664, -434] | -152[-406, 17] | <0.001 |
| CTR | 0.00[0.00, 0.00] | 0.00[0.00, 0.00] | <0.001 | 0.00[0.00, 0.00] | 0.00[0.00, 1.00] | <0.001 |
| **Change after Follow-up** |  |  |  |  |  |  |
| MD growth (mm) | / | / | / | 2.9[2.2, 3.6] | 4.4[3.0, 7.3] | <0.001 |
| MD growth in ratio (%) | / | / | / | 30[23, 50] | 40[22, 68] | 0.280 |
| AD growth (mm) | / | / | / | 2.0[1.5, 2.8] | 3.9[2.3, 6.3] | <0.001 |
| AD growth in ratio (%) | / | / | / | 30[15, 40] | 30[17, 62] | 0.006 |
| VDT (days) | / | / | / | 525[316, 805] | 326[166, 633] | 0.001 |
| CT value increase (HU) | 15[-19, 56] | 25[-11, 82] | 0.012 | 40[4, 113] | 57[4, 139] | 0.403 |
| **Patient Characteristic** |  |  |  |  |  |  |
| Age (years) | 56[47, 63] | 62[57, 67] | <0.001 | 63(9) | 63(8) | 0.992 |
| Gender |  |  | <0.001 |  |  | 0.001 |
| Male | 119(20%) | 73(36%) |  | 23(26%) | 93(49%) |  |
| Female | 462(80%) | 127(64%) |  | 64(74%) | 98(51%) |  |
| Ever smoker |  |  | <0.001 |  |  | 0.004 |
| No | 557(96%) | 172(86%) |  | 83(95%) | 159(83%) |  |
| Yes | 24(4%) | 28(14%) |  | 4(5%) | 32(17%) |  |
| Height (cm) | 160[157, 165] | 162[158, 169] | 0.009 | 160[156, 166] | 164[159, 170] | 0.001 |
| Weight (kg) | 59[53, 66] | 60[55, 70] | 0.022 | 60[54, 66] | 62[56, 70] | 0.062 |
| BMI (kg/mm2) | 23.02(2.88) | 23.37(3.30) | 0.152 | 23.53(2.92) | 23.49(3.18) | 0.916 |

Data are n(%), median[IQR] or X(SD). P for Mann-Whitney U-test, chi-squared test, Fisher's exact test, or Independent sample t-test.

e-Table 2: The univariate and multivariate analysis of predicting malignancy in stable nodules.

| **Characteristics** | **Identification of Lung Cancer in Stable Nodules** | | | | | |
| --- | --- | --- | --- | --- | --- | --- |
|  | **Univariate Logistic Regression** | | | **Multivariate Logistic Regression** | | |
|  | **β** | **Odds Ratio(95%CI)** | **P** | **β** | **Odds Ratio(95%CI)** | **P** |
| **Nodule Type** |  |  |  |  |  |  |
| PGGN | 0.000 | 1 | <0.001 |  |  |  |
| MGGN | -1.205 | 0.300(0.176~0.511) | <0.001 |  |  |  |
| SN | -3.001 | 0.050(0.028~0.089) | <0.001 |  |  |  |
| **Nodule Location** |  |  |  |  |  |  |
| Left upper lobe | 0.000 | 1 | 0.003 |  |  |  |
| Left lower lobe | -1.246 | 0.288(0.145~0.569) | <0.001 |  |  |  |
| Right upper lobe | -0.500 | 0.607(0.327~1.127) | 0.113 |  |  |  |
| Right median lobe | -0.727 | 0.484(0.210~1.115) | 0.088 |  |  |  |
| Right lower lobe | -1.034 | 0.356(0.178~0.709) | 0.003 |  |  |  |
| **Baseline CT** |  |  |  |  |  |  |
| Maximum Diameter (>7.4mm) | 0.683 | 1.979(1.308~2.997) | 0.001 |  |  |  |
| Vertical Maximum Diameter (>7.1mm) | 0.813 | 2.255(1.448~3.511) | <0.001 |  |  |  |
| Nodule Thickness (>7.6mm) | 0.818 | 2.267(1.494~3.440) | <0.001 |  |  |  |
| Average Diameter (>7.2mm) | 0.745 | 2.105(1.391~3.187) | <0.001 | 1.259 | 3.521(2.117~5.856) | <0.001 |
| CT value (>-478HU) | -1.801 | 0.165(0.105~0.259) | <0.001 | -0.878 | 0.415(0.220~0.784) | 0.007 |
| CTR (>0.78) | -2.740 | 0.065(0.037~0.112) | <0.001 | -2.047 | 0.129(0.064~0.262) | <0.001 |
| **Preoperative CT** |  |  |  |  |  |  |
| CT value (>-305HU) | -2.067 | 0.127(0.082~0.196) | <0.001 | -0.803 | 0.448(0.228~0.880) | 0.020 |
| CTR (>0.79) | -2.740 | 0.065(0.037~0.112) | <0.001 |  |  |  |
| **Patient Characteristic** |  |  |  |  |  |  |
| Weight (>61kg) | -0.584 | 0.558(0.367~0.848) | 0.006 |  |  |  |
| BMI (>22.77kg/mm2) | -0.777 | 0.460(0.295~0.717) | 0.001 |  |  |  |

Only significant risk factors were listed.
